# Treatment with eucalyptol mitigates cigarette smoke-induced lung injury through suppressing ICAM-1 gene expression

**DOI:** 10.1042/BSR20171636

**Published:** 2018-07-06

**Authors:** Na Yu, Yi-Tian Sun, Xin-Ming Su, Miao He, Bing Dai, Jian Kang

**Affiliations:** 1 Department of Respiratory and Critical Care Medicine, Institute of Respiratory Diseases, The First Hospital of China Medical University, Shenyang, Liaoning 110001, China; 2 Department of Respiratory Medicine, Affiliated Hospital of Xuzhou Medical University, Xuzhou, Jiangsu 221002, China; 3 Department of Environmental and Health,School of Public Health, China Medical University, Shenyang, Liaoning 110122, China

**Keywords:** cigarette smoke, cytokines ICAM-1, Eucalyptol, inflammatory cells, lung function

## Abstract

The present study was conducted to investigate the clinical significance of Eucalyptol in treating cigarette smoke-induced lung injury with the potential mechanism involved in the event. Rats were exposed to air (control) and cigarette smoke (smoking) after they were treated with Eucalyptol (260 mg/kg) orally once a day for 12 weeks. Cell counts of bronchoalveolar lavage fluid (BALF), measurements of mean liner intercept (MLI) and mean alveolar number (MAN), and lung function test were executed in experimental animals. Contents of cytokines and intercellular adhesion molecule (ICAM)-1 in BALF and ICAM-1 protein and mRNA expression in lung tissues were determined by ELISA, immunohistochemistry (IHC), and RT-PCR, respectively. A rat model of chronic obstructive pulmonary disease (COPD) displayed declining lung function, increased cell counts and cytokine production in BALF, and emphysema-like lesions in cigarette smoke-exposed lungs compared with the controls (all *P*<0.01). Treatment with Eucalyptol partly reversed lung function decline with obvious decrease in inflammatory cell infiltrate, TNF-α, IL-6, and ICAM-1 expression levels in the challenged lungs (all *P*<0.05 and 0.01). Furthermore, oral administration of the drug not only reduced the emphysema-associated lung lesions but also suppressed ICAM-1 protein and mRNA expression in the lungs compared with the control (all *P*<0.05 or 0.01). Intervention of Eucalyptol mitigates the ongoing inflammatory process in airways and ameliorates the cigarette smoke-induced lung injury through suppressing ICAM-1 gene expression in the diseased lungs.

## Introduction

Chronic obstructive pulmonary disease (COPD) is a preventable and treatable disease that is characterized by persistent airway inflammation and airflow limitation in the disease process [1,2]. Smoking is the primary cause of COPD and leads to an airway inflammatory response [3,4]. Moreover, there is a higher incidence of respiratory symptoms and lung function abnormalities in smokers compared with non-smokers [5]. Morbidity and mortality due to COPD increase worldwide [6,7], whereas it is estimated that this disorder will become the third leading cause of global deaths by 2020 [8]. Accordingly, an animal model of COPD requires to be established for identifying the effects of therapeutic drugs on the disease [9–11]. Though there are plenty of drugs that have been used to treat COPD in clinical practice, it is still lacking in rescuing and controlling the disease progression.

Eucalyptol is a natural organic compound of many plant essential oils, mainly extracted from Eucalyptus oil [12]. This compound, a colorless liquid, is traditionally used to treat respiratory disorders due to its secretolytic and anti-inflammatory properties in the airways [13]. Although intervention of Eucalyptol showed some benefits for COPD patients [14], the mechanism of the action of the drug remains largely unclear.

The present study was to investigate the clinical significance and the mechanism of Eucalyptol in treating cigarette smoke-associated lung injury. Our results revealed that this natural organic compound not only mitigates the ongoing inflammatory response in airways but also relieves the lung injury through suppressing intercellular adhesion molecule (ICAM)-1 gene expression in the diseased lungs.

## Materials and methods

### Animals and exposure to environmental tobacco smoke

Male 12-week-old Sprague–Dawley rats weighing approximately 200 g were purchased from the Experimental Centre of Animals at China Medical University. The animals were housed in an environmentally controlled animal facility of our hospital for the duration of the experiments. All procedures were reviewed and approved by the Research Review Committee of The First Affiliated Hospital of China Medical University.

Animals were randomly divided into four groups of six each. The protocol for making the animal model of cigarette smoke-induced COPD was modified with different approaches during a smoking period. Da qian men tobacco cigarettes used for challenging animals were commercially purchased from Shanghai Tobacco (Group) Corporation (Shanghai, China). Rats were confined in a chamber (80 × 60 × 58cm) with two holes at the top (outlet) and the low part of the chamber (input) connected to a small air pump by a flexible hose. The animal model was accomplished with mainstream smoke exposure to tobacco smoke (16 cigarettes for 30 min twice daily) for 12 consecutive weeks in a cigarette smoke chamber [15,16]. The concentration of Eucalyptol was selected in reference to other studies [17,18]. Eucalyptol (Johamu Pharmaceutical Co., Ltd. Beijing, China) was prepared at the dose of 260 mg/kg dissolved in 10% glycerin. Rats were treated with this compound orally (once a day × 12 weeks) 30 min prior to smoking. An equivalent volume and concentration of glycerin was given as a comparative treatment. Rats in the control group were exposed to air in the same procedure of the experiments.

### *In vivo* responsiveness

Rats were anesthetized by an intraperitoneal injection of 1% pentobarbital sodium (40 mg/kg) and maintained with an appropriate plane of the anesthesia. A ratio of forced expiratory volume (FEV) at 0.3 s (FEV0.3) and forced vital capacity (FVC), and functional residual capacity (FRC) were examined by using a differential pressure transducer to measure the rat’s respiration in an animal plethysmograph. Briefly, the rat trachea was opened with an inverted T-shaped incision in the position between the second and the third cartilage ring, rapidly intubated, and attached to a ventilator (Res3020, Bestlab High-Tech Co., Ltd., Beijing, China) at a respiratory rate of 75 beats per min with a low tidal volume (5 ml/kg). FEV0.3/FVC and FRC were determined using data automatically obtained with the AniRes2005 pulmonary mechanics analyzer (version 3.0, Bestlab High-Tech Co., Ltd., Beijing, China).

### Cell count and preparation of sample

Rat lungs were lavaged by instillation and withdrawal of 4.0 ml PBS (five times) through a tracheal cannula, and an equal volume of bronchoalveolar lavage fluid (BALF) was collected from individual rats. The BALF sample from each rat was centrifuged (1200 r.p.m. × 10 min) at 4°C and supernatant was stored at −80°C for the ELISA. The total BALF cells were counted using a hemocytometer. The BALF cells from each sample were applied to a glass slide using a cytospin (1200 r.p.m. × 10 min) and then the slide was stained with Hema 3 Stain Set (Fisher Scientific, U.S.A.) for the differential count of cells. The relative proportion of different cells was determined by counting 300 cells/slide and then was factored to the number (×10^3^/ml) of total BALF cells collected in each group.

A part of right lung tissues was removed from the rats and immediately snap-frozen in liquid nitrogen. The samples were stored at −80°C for RT-PCR analysis.

### Determination of cytokine and ICAM-1 production

Values of optical densities (ODs) for the specific protein contents in BALF were examined using ELISA kits (Dakewe Biotech Co., Ltd, Beijing and Abcam Cambridge, MA) after a total protein concentration was measured by BCA assay. The OD value of the protein level was calculated using the formula: OD value = Background OD − measured value (an average value from five tests). The TNF-α, IL-6, and ICAM-1 production in the samples was determined using antibodies against the murine TNF-α (DKW12-3720), IL-6 (DKW12-3060), and ICAM-1 (ab33894, Abcam) according to the manufacturer’s directions. Briefly, 100 µl substrate solution was added to 100 µl sample per well in a microtiter plate. One hundred microlitres of biotinylated antibody (1:100) was introduced to each well and then the mixture was incubated for 90 min at 37°C. The plate was added with streptavidin-HRP (1:100) per well after washing four times with PBS and incubated for 30 min at 37°C. The enzyme-substrate reaction was terminated by adding 100 µl of 4 M sulphuric acid, and the OD values were read in a microtiter autoreader at 450 nm.

### Morphological study

Mean liner intercept (MLI) in lung tissue and mean alveolar number (MAN) were calculated in morphological analysis. MLI was determined for each region studied on an overlay consisting of horizontal and vertical lines. All intercepts with alveolar septal number (ASN) were counted at the intersection point of the two lines in the central field of the view under microscope. The total length (L) of all the lines together divided by the number of intercepts gives the mean linear intercept for the region studied. A formula shown as MLI = L/ASN (μm), which is used to estimate an average diameter of a single alveolus in size. MAN was determined according to alveolar number (AN) in each field of view and a square area (SA) of the field. A formula is shown as MAN = AN/SA (number/μm^2^), which is an indicator for density of alveoli.

### Immunohistochemistry staining

Specimens of left lung were taken from the animals. Each one was cut into three pieces juxtaposed to each other and of equal size. The middle part was inflated in PBS containing 4% (v/v) formalin under vacuum (13 kPa) for 20 min using a routine water stream-driven device (water aspirator). Sections (3 μm) were cut, and were taken care to prevent overstretching before they were deparaffinized, dehydrated, and subjected to antigen retrieval. Immunohistochemical detection for ICAM-1 expression in the rat lung was carried out using immunohistochemistry (IHC) kit (Abcam, Cambridge, MA) according to the manufacturer’s recommendations. Briefly, the sections were incubated with a primary antibody (ab33894, Abcam) at a concentration of 0.5 μg/ml overnight at 4°C. The following day, the sections were incubated with HRP-conjugated secondary antibody for 1 h at 37°C. After incubating with DAB/H_2_O_2_, the sections were counterstained with Hematoxylin and mounted for identifying ICAM-1 expression under a light microscope at 400× magnification (Olympus, Japan). Five circular areas from high power fields on each slide were selected according to the color response. The images of ICAM-1 expression for immunostaining were captured by SPOT Advanced™ software (Modular Imaging Software for Microscopy, U.S.A.). Integrated OD (IOD) of ICAM-1 in each field was examined by using microimage analysis system Metamorph/Evolution MP5.0/BX51 (US/JP, UIC/Olympus). The mean value of IOD for every five fields was considered as the measured value (mean ± S.D.) for every section. The ICAM-1 protein concentration in rat lungs was quantitated by IOD (area of the measured object × mean OD).

### Determination of *ICAM-1* mRNA

*ICAM-1* mRNA expression levels in lung tissues were assessed by using SYBR Premix Ex Taq™ (Takara, Japan) and mRNA levels were normalized to GAPDH housekeeping gene. The following forward primers for the *ICAM-1* mRNA and GAPDH were used sequences in the 5′-GCTTCTGCCACCATCACTGTGTA-3′ and 5′-GCAAGTTCAACGGCACA-3′. Reverse primers for ICAM-1 and GAPDH were 5′-ATGAGGTTCTTGCCCACCTG-3′ and 5′-CATTTGATGTTAGCGGGAT-3′. Briefly, the total content of RNA in the tissues was extracted using a tissue homogenizer in Qiagen lysis buffer and purification of RNA was performed with Qiagen RNeasy minicolumns following the manufacturer’s protocol. RNA was quantitated using the NanoDrop ND-1000 spectrophotometer and amplified and biotin-labeled with Nugen’s Ovation System, according to the manufacturer’s instructions (LightCycler 480, Roche, Switzerland). The yield of the total RNA per replicate varied from 0.6 to 2.0 μg. Fifty nanograms of the RNA was added in a SYBR qPCR Master Mix for real-time RT-PCR. Quantitative data of *ICAM-1* mRNA after normalizing to GAPDH were shown with a fold-change compared with the expression level of the control sample.

### Statistical analysis

Data were expressed as mean ± S.D. in the results. Statistical analysis was performed using Statistical Package for the Social Science (SPSS, version 16.0) since an assessment of the normality of data is a prerequisite for many statistical tests. Comparisons from groups were performed by one-way ANOVA based on the consideration that this technique can often be used for numerical response data. Student’s paired *t* test was used to compare measurements of individual groups. *P*-values less than 0.05 were considered significant.

## Results

### Establishment of COPD model

**Figure 1:**
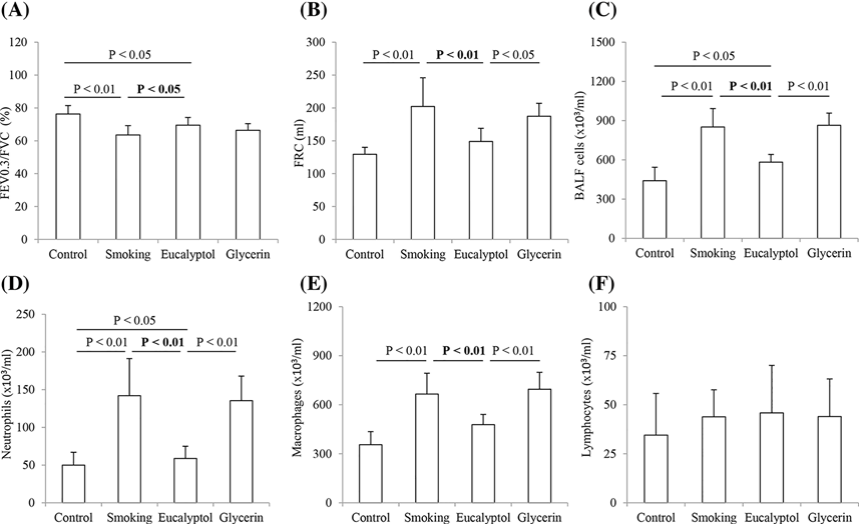
Lung function test and cell count in BALF Rats were exposed to air (control) and cigarette smoke (smoking) in presence and absence of Eucalyptol. FEV0.3/FVC was decreased with an increased FRC in smoking rats (**A**,**B**). Treatment with Eucalyptol partly reversed the damage in lung function in smoking rats. The numbers (×10^3^/ml) of total cells, neutrophils, macrophages but not lymphocytes in the BALF samples from the rats exposed to cigarette smoke were enhanced (**C**–**F**). Intervention of Eucalyptol reduced the numbers of total cells, neutrophils, and macrophages in BALF of the cigarette smoke-exposed rats. Data were expressed as mean ± S.D. (*n*=6).

Animal model of COPD was initially identified according to the changes in lung function and airway inflammation and the results are shown in Figure 1. FEV0.3/FVC was significantly decreased with an increased FRC in the rats exposed to cigarette smoke compared with the control animals (both *P*<0.01, *n*=6). Treatment with Eucalyptol but not glycerin partly reversed the changes in FEV0.3/FVC and FRC in smoking rats (Figure 1A,B). Average values for FEV0.3/FVC (%) and FRC (ml) were 76.35 ± 5.09 and 129.57 ± 10.46 in the control group, 63.57 ± 5.68 and 202.35 ± 43.64 in smoking rats, 69.50 ± 4.68 and 148.96 ± 20.03 in the Eucalyptol group, and 66.37 ± 4.11 and 187.45 ± 19.54 in the glycerin group. In contrast, there was a statistical difference in FEV0.3/FVC between the two groups of Eucalyptol-treated rats and either the control or cigarette smoke-exposed animals (both *P*<0.05). Furthermore, there was statistically significant difference in FRC between the two groups of Eucalyptol-treated rats and either cigarette smoke-exposed or glycerin-treated animals (*P*<0.01 and 0.05).

Cells in the BALF samples from the grouped rats were counted in presence and absence of Eucalyptol (Figure 1C–F). The average values (×10^3^/ml) for the numbers of the total cells, neutrophils, macrophages, and lymphocytes were shown as 439.83 ± 103.14, 50.0 ± 16.92, 355.33 ± 80.14, and 34.5 ± 21.26 in the control group, 851.67 ± 139.79, 142.0 ± 49.15, 665.33 ± 127.44, and 43.83 ± 13.72 in the smoking group, 582.0 ± 59.2, 58.67 ± 16.33, 477.33 ± 63.28, and 45.83 ± 24.22 in the Eucalyptol group, and 864.33 ± 93.37, 135.33 ± 32.64, 694.67 ± 103.14, and 44.0 ± 19.13 in the glycerin group, respectively. Inflammatory cell infiltrate was very obvious with significant increase in the counts of neutrophils and macrophages but not lymphocytes in the samples from both cigarette smoke-exposed rats and glycerin-treated animals. Treatment with Eucalyptol significantly reduced the total and differential cell counts in BALF of smoking rats with statistical differences in the counts of total cell, neutrophils, and macrophages between cigarette smoke-exposed rats and either the control or Eucalyptol-treated animals (*P*<0.01; *n*=6). Additionally, there were statistically significant differences in the counts between Eucalyptol-treated rats and either the control or glycerin-treated ones (*P*<0.05 or 0.01).

### Cytokine and ICAM-1 content

**Figure 2:**
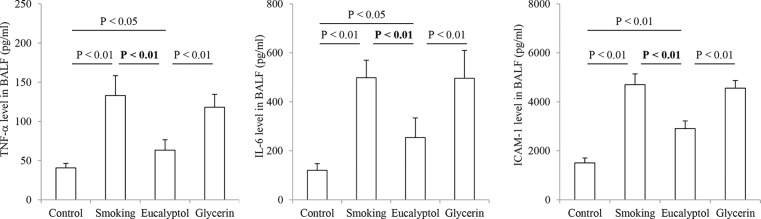
Contents of TNF-a, IL-6, and ICAM-1 in BALF Contents (pg/ml) of TNF-α, IL-6, and ICAM-1 protein productions in BALF were determined in rats exposed to air (control) and cigarette smoke (smoking) in presence and absence of Eucalyptol. Data were expressed as mean ± S.D. (*n*=6).

TNF-α, IL-6, and ICAM-1 protein levels in the BALF samples from experimental rats were examined in the presence and absence of Eucalyptol and the results are shown in Figure 2. Average levels (pg/ml) of TNF-α, IL-6, and ICAM-1 proteins were indicated as 40.74 ± 5.89, 120.74 ± 27.42, and 1058.63± 200.33 in the control group, 132.99 ± 25.46, 498.74 ± 71.45, and 4701.54 ± 440.12 in smoking rats, 63.33 ± 13.46, 254.37 ± 79.68, and 2908.05 ± 314.1 in the Eucalyptol group, and 118.08 ± 16.52, 496.59 ± 113.6, and 4457.9 ± 315.97 in the glycerin group. All the proteins’ contents in the samples of smoking rats were significantly increased compared with those of the control (all *P*<0.01). Treatment with Eucalyptol but not glycerin obviously decreased the contents of TNF-α, IL-6, and ICAM-1 proteins in BALF of smoking rats. In contrast, there were statistical differences in the protein levels between cigarette smoke-exposed rats and either the control or Eucalyptol-treated animals (all *P*<0.01; *n*=6). Additionally, there were significant differences in the protein levels between Eucalyptol-treated rats and either the control or glycerin-treated animals (all *P*<0.05 or 0.01).

### Size and number of alveoli

**Figure 3:**
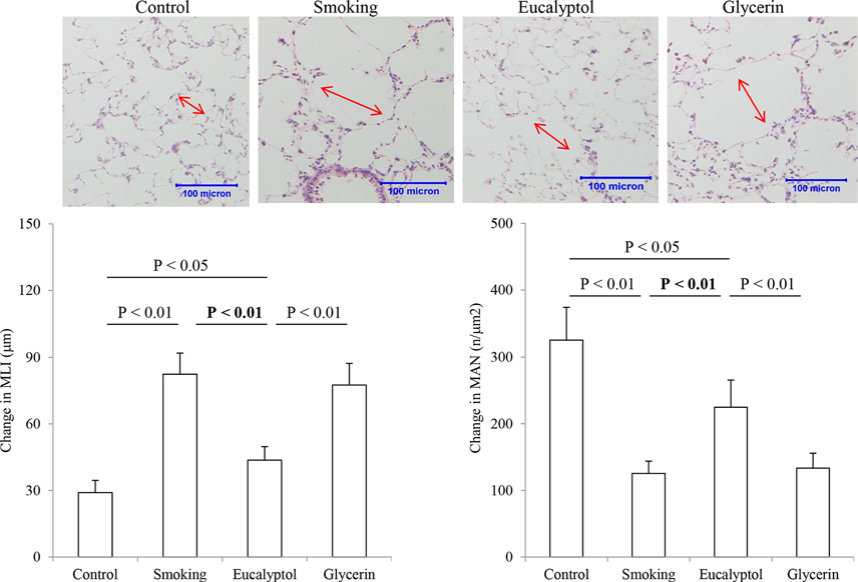
Morphological features of lung structures Pathological changes in micro-anatomy of lung tissues were examined in rats exposed to air (control) and cigarette smoke (smoking) in presence and absence of Eucalyptol. Images regarding the changes in microanatomy in lung tissues were taken with arrows (red) to show the areas measured for morphometric analysis (upper). MLI and MAN were measured in microscopic vision of the lungs (down). Data were expressed as mean ± S.D. (*n*=6).

Structural features of cigarette smoke-challenged lungs were examined using MLI and MAN measurement technique and the results are shown in Figure 3. Morphologically, smoking rats showed an enlargement of alveolar airspaces and destruction of septal walls of alveoli in the field of the view under microscope (×400 magnification) compared with the control. Treatment with Eucalyptol but not glycerin may reduce the lung injuries confirmed by visual observation. Average values of MLI (μm) and MAN (number/μm^2^) were 29.09 ± 5.50 and 325.42 ± 49.45 in the control, 82.47 ± 9.51 and 125.48 ± 18.35 in smoking rats, 43.68 ± 6.09 and 224.68 ± 40.81 in the Eucalyptol group, and 77.56 ± 9.77 and 133.54 ± 22.32 in the glycerin group. The enlargement of air spaces and decrease in alveolar density were clearly observed in the tobacco smoke-exposed lungs as compared with the control (both *P*<0.01; *n*=6). Treatment with Eucalyptol significantly mitigated the lung injuries. In contrast, there were statistical differences in measurements of MLI and MAN between smoking rats compared with either the control or Eucalyptol-treated animals, and Eucalyptol-treated rats compared with either the control or glycerin-treated animals (*P*<0.05 or 0.01).

### ICAM-1 protein expression in lungs

**Figure 4:**
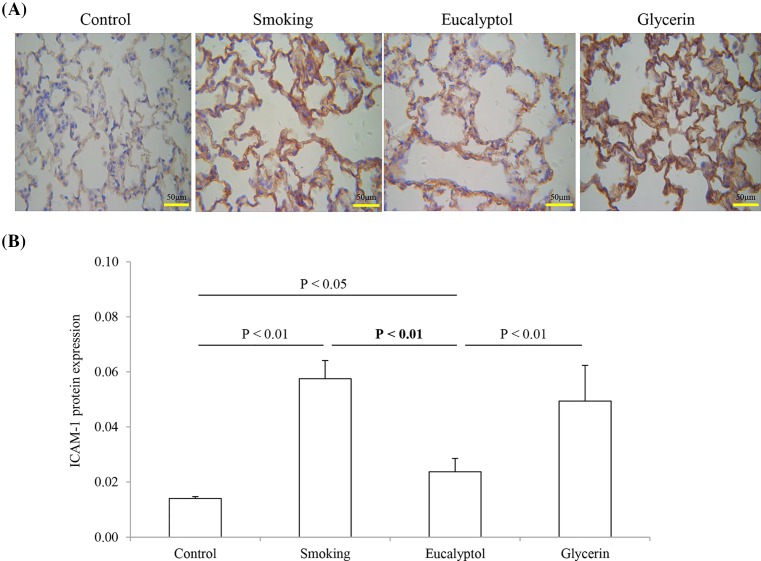
ICAM-1 protein expression Rats were exposed to air (control) and cigarette smoke (smoking) in presence and absence of Eucalyptol. ICAM-1 expression (**A**) in lung tissues was observed by the IHC method. Relative OD (**B**) for ICAM-1 protein expression in the tissues was expressed as mean ± S.D. (*n*=6) in each group.

ICAM-1 protein expression in lung tissues was examined using an IHC method in rats exposed to air and cigarette smoke in presence and absence of Eucalyptol. The results are shown in Figure 4. A positive response of ICAM-1 protein to its antibody may be observed in the lung tissues stained in brown color (Figure 4A). Appearance of the protein was overexpressed in the tissue samples from the smoking rats and the glycerin-treated rats as compared with these from the control and the Eucalyptol-treated ones.

Relative IOD for ICAM-1 protein expression in the lung tissues was calculated in the grouped animals (Figure 4B). Average values for the changes in the protein density were 0.014 ± 0.0007 in the control group, 0.058 ± 0.0066 in smoking rats, 0.024 ± 0.0049 in the Eucalyptol group, and 0.050 ± 0.0130 in the glycerin group. The value for the change of the density significantly increased in smoking rats. Treatment with Eucalyptol but not glycerin obviously reduced the protein expression in the smoked lungs. In statistical analysis, there were significant differences in ICAM-1 protein expression between smoking compared with either the control or the Eucalyptol group (both *P*<0.01; *n*=6). There were obvious differences in the protein expression levels between the Eucalyptol group compared with either the control or the glycerin group (*P*<0.05 or 0.01).

### *ICAM-1* mRNA expression in lungs

**Figure 5:**
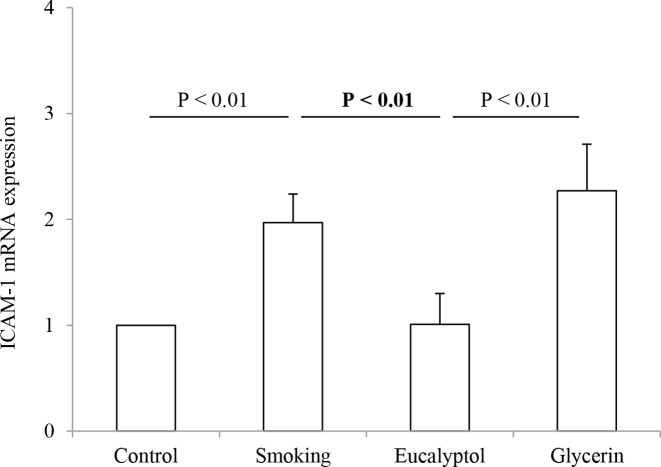
*ICAM-1* mRNA expression *ICAM-1* mRNA expression in the lung samples were determined in rats exposed to air (control) and cigarette smoke (smoking) in presence and absence of Eucalyptol. The mRNA expression level in the samples was calculated as a fold change in reference to the control. Data were expressed as mean ± S.D. (*n*=6) in each group.

*ICAM-1* mRNA expression in lungs was determined in rats exposed to air and cigarette smoke in presence and absence of Eucalyptol. The results are shown in Figure 5. *ICAM-1* mRNA expression in lung tissues was calculated as a fold change in reference with the expression level of the control mRNA. The *ICAM-1* gene was up-regulated in cigarette smoke-exposed lungs with 1.97- and 1.95-fold increase over the control and the Eucalyptol-treated animals. The average values for the gene expression levels were shown as 1.00 ± 0.00 in the control group, 1.97 ± 0.27 in smoking rats, 1.01 ± 0.29 in the Eucalyptol group, and 2.27 ± 0.44 in the glycerin group. Treatment with Eucalyptol but not glycerin resulted in a significant decrease in the gene expression level. In contrast, there were significant differences in the expression levels of the gene product between smoking compared with either the control or the Eucalyptol group (both *P*<0.01; *n*=6)). Additionally, there was a statistical difference between the treatments with Eucalyptol and glycerin (*P*<0.01).

## Discussion

Studies have provided the evidence that COPD is characterized by airflow limitation and an abnormal inflammatory response in the lungs [19,20]. In initial experiments, a rat model of COPD was established based on the observation that a 12-week cigarette smoke challenge was able to model the abnormal lung function of the animals [21,22]. Our results show that this model is associated with a significant decrease in FEV0.3/FVC and an obvious increase in FRC, indicating existence of tobacco smoke-related airflow obstruction. In association with declining lung function, a large number of inflammatory cells, comprise mainly neutrophils and macrophages, into the BALF may be found, indicating an airway inflammatory response to tobacco smoke inhalation in the rat model of COPD because mechanical ventilation with low tidal volume does not alter pulmonary inflammatory responses [23–25]. Treatment with Eucalyptol not only partly improved lung function but also significantly reduced the numbers of neutrophils and macrophages in BALF of smoking rats, suggesting that this compound ameliorated lung function decline through inhibiting ongoing inflammatory responses since airflow obstruction typically in COPD is related to progressive airway inflammation [26,27]. Eucalyptol is a natural organic compound which has therapeutic benefits in COPD due to its antioxidative and anti-inflammatory properties [12,28]. Although clinical benefits of Eucalyptol applied for COPD patients are still being explored [14,29], the effects of the agent on reducing cigarette smoke-driven airway inflammation are given explicit support in treating the disease. Furthermore, our results also provide the promise that airflow limitation in COPD is partially reversible with anti-inflammatory therapies [30,31].

TNF-α, IL-6, and ICAM-1 protein contents were examined in the BALF samples from experimental animals. In contrast with the control, smoking rats showed abundance of TNF-α, IL-6, and ICAM-1 productions in the samples. These finding were completely consistent with the results of inflammatory cell infiltrate, which supported the conclusion that the enhanced cytokine productions in the challenged lungs were caused mainly due to inflammatory cell activation considered a prominent feature in COPD. Furthermore, the high levels of TNF-α and IL-6 productions in COPD were associated with impaired lung function [32]. The increase in ICAM-1 content referred to neutrophil infiltration in the inflammatory process since the adhesion molecule was often involved in a number of inflammatory processes such as neutrophil trafficking [33]. These findings were identical to the clinical studies in which COPD patients exhibit heightened airway inflammation, characterized by the increased levels of proinflammatory cytokines such as TNF-α and IL-6 and by a predominantly neutrophilic cellular infiltrate in the airway lumen [34–36]. Treatment with Eucalyptol but not glycerin resulted in obviously lowering the levels of TNF-α, IL-6, and ICAM-1 productions, suggesting that the dose of this compound used in the model was sufficient and effective for reducing the release and expression of the inflammatory cell-derived proteins. Since Eucalyptol displayed potent anti-inflammatory property, it is conceivable that this compound would play a role in protecting lung tissues against the inflammation-inducing injuries.

Pathologic features of micro-anatomy of cigarette smoke-exposed lungs were observed using morphometric techniques that have been involved in the direct and unbiased estimation for the quantitative analysis of lung injuries [37–39]. Our results showed that the values for measurements of MLI obviously increased with the reduced MAN in cigarette smoke-exposed lungs as compared with the control, indicating the emphysema-like changes including enlargement of distal air space in size and reduction in alveolar density in the animal model of COPD. Treatment with Eucalyptol but not glycerin mitigated the lung injuries, so it led us to speculate the potential efficacy of this compound in rescuing and alleviating morphological deficits in lungs through attenuating the intensity of the inflammatory response in the diseased lungs.

ICAM-1 participates in trafficking of inflammatory cells and its expression level is regulated by cytokines [33,40]. Our results showed that the protein expression in airway epithelial cells markedly increased in the smoking rats as compared with the control animals, suggesting that airway inflammatory process required ICAM-1 involvement in the model. Intervention of Eucalyptol but not glycerin obviously decreased the protein expression level in the challenged lungs, indicating that this compound reduced the inflammatory response by altering the adhesion molecule expression level. It has been reported that neutrophil recruitment to lungs is significantly reduced in mice with anti-ICAM-1 antibody [41]. Moreover, ICAM-1 is up-regulated in the response to a number of inflammatory mediators such as TNF-α and IL-6 [42]. Therefore, the protein should be considered as a therapeutic target that, when blocked by Eucalyptol, inhibit the excessive transmigration of neutrophils with cytokines released from the inflamed airways and hence limit the inflammatory process in COPD.

In agreement with ICAM-1 protein overexpression, its mRNA expression level in the challenged lung tissues was increased with a 1.97-fold increment over the control. Treatment with Eucalyptol resulted in obvious suppressing the gene expression with a 1.95-fold decrease as compared with the smoking rats treated with and without glycerin, confirming a molecular mechanism contributing to the role of Eucalyptol in the diseased lungs. ICAM-1 gene was up-regulated in COPD patients [43], whereas the increase in the gene expression was related to sustained recruitment of neutrophils into the inflamed airway tissue, which in turn led to structural destruction in emphysema [44,45]. Based on the facts that Eucalyptol suppressed expression of the *ICAM-1* gene in the smoked lungs, this compound showed a high merit in protecting lungs against the injuries by the on-going airway inflammatory process.

In conclusion, intervention of Eucalyptol not only reduces the inflammatory cell infiltrate and the release of proinflammatory cytokines but also mitigates functional and structural damages of the rat lungs exposed to cigarette smoke through suppressing ICAM-1 gene expression in the target tissues.

## Abbreviations

ASN: alveolar septal number
BALF: bronchoalveolar lavage fluid
COPD: chronic obstructive pulmonary disease
DAB: diaminobenzidine
FEV: forced expiratory volume
FEV0.3: FEV at 0.3 s
FRC: functional residual capacity
FVC: forced vital capacity
GAPDH: glyceraldehyde-3-phosphate dehydrogenase
HRP: horseradish peroxidase
ICAM: intercellular adhesion molecule
IHC: immunohistochemistry
IOD: integrated optical density
IL-6: interleukin-6
MAN: mean alveolar number
MCh: methacholine
MLI: mean liner intercept
OD: optical density
RT-PCR: reverse transcription-polymerase chain reaction
SA: square area
TNF-α: tumor necrosis factor-α
